# Perforator Versus Non-perforator Flap for Perineal Reconstruction: Long-Term Comparison of Complications and Quality of Life

**DOI:** 10.1245/s10434-026-19815-7

**Published:** 2026-06-06

**Authors:** Séverin R. Wendelspiess, Josefin T. Sandstedt, Julia Stoffel, Nadia Menzi, Maximilian Burger, Florian S. Halbeisen, Ioana Lese, Tarek Ismail, Adriano Fabi, Loraine Kouba, Dirk J. Schaefer, Elisabeth A. Kappos

**Affiliations:** 1 Department of Plastic, Reconstructive, Aesthetic and Hand Surgery, University Hospital Basel, Basel, Switzerland; 2 Faculty of Medicine, University of Basel, Basel, Switzerland; 3 Faculty of Medicine, University of Bern, Bern, Switzerland; 4 Department of Plastic and Hand Surgery, Inselspital University Hospital Bern, Bern, Switzerland; 5 Department of Clinical Research, Surgical Outcome Research Center, University Hospital Basel and University of Basel, Basel, Switzerland

**Keywords:** Perforator flap, Musculocutaneous flap, Perineal reconstruction, PROMs, Quality of life, Complications

## Abstract

**Background:**

Perineal reconstruction remains challenging due to complex anatomy, high bacterial load, and frequent postoperative complications. Extensive tissue defects after oncologic resection or severe infection commonly necessitate reconstructive procedures. Although myocutaneous flaps are widely used, perforator flaps offer advantages such as muscle preservation and reduced donor-site morbidity. Given the perineal region’s critical role in defecation, urination, and sexual function, evaluating patients’ quality of life (QoL) after reconstructive surgery is essential.

**Methods:**

This single-center cohort study evaluated postoperative complication rates and QoL after perineal reconstruction with perforator versus non-perforator flaps from 2013 to 2023. All participants were invited to complete a postoperative QoL survey.

**Results:**

Of all the patients, 58 % received a perforator-based and 40 % a non-perforator-based reconstruction. One patient (2.3 %) underwent a combined approach. The primary indication for perineal reconstruction (68.9 %) was defect coverage after oncologic resection. Both groups had a 50 % complication rate. Donor-site morbidity was higher in the non-perforator flap group, with all complications classified as Clavien-Dindo grade III. Additional findings were exploratory: patient satisfaction was higher in the non-perforator flap group (100 % vs 66 %), although this group had a substantially longer follow-up period (4.14 vs 1.74 years). Conversely, numerically higher QoL scores were observed in the perforator flap group.

**Conclusion:**

Perforator flaps demonstrated a more favorable donor-site profile, representing the most robust finding of this cohort. Observed differences in complications and patient-reported outcome measures are exploratory, and QoL continues to be insufficiently addressed in oncologic perineal reconstruction, underscoring the need for enhanced interdisciplinary collaboration.

The management of oncologic, infectious, and inflammatory diseases of the perineal region frequently results in extensive and complex defects in this functionally demanding anatomical region.^1–4^ In females, the proximity of the vagina, urethra, and anus to the surgical defect and the resulting high bacterial colonization challenge optimal wound-healing.^5^ Consequently, reconstructive surgery constitutes a pivotal aspect in the effective management of these patients, with the objective of restoring structural integrity and preserving essential functions such as urination, defecation, and sexuality.^6^

Classic flap-based reconstructive methods in the perineal region often include non-perforator flaps.^7–9^ Non-perforator flaps are valid options for patients requiring robust tissue coverage of large defects with large dead space volume. Examples for these non-perforator flaps are local, fasciocutaneous flaps (e.g., posterior thigh flap), and musculocutaneous flaps (e.g., vertical rectus abdominis musculocutaneous [VRAM] flap, gracilis flap).^10–13^ However, non-perforator flaps are associated with a high incidence of complications at the donor site, and in cases of a muscle-based flap reconstruction, there is a potential risk for loss of function at the donor site.^14–16^ To minimize this donor-site morbidity, there has been a noted increase in the use of perforator flaps in recent years. These have become the gold standard in reconstructive surgery (e.g., in autologous breast surgery).^17–19^

Perforator flaps are based on small perforator vessels that originate from deeper arteries. These perforator vessels reach through the muscle or fascia and supply an “angiosome,” which is a specific area of subcutaneous tissue together with the overlying skin, allowing the underlying muscle to be preserved, consequently lowering donor-site morbidity.^20,21^

Evaluation of the impact that surgical treatments has on patients’ quality of life (QoL) using validated patient-reported outcome measures (PROMs) has become increasingly important.^22,23^ For clinicians, PROMs offer valuable insights into patient experiences by capturing their perspectives of recovery, overall satisfaction, and challenges that they face after surgery, which have historically been underreported in surgical outcome research. Patient satisfaction is a critical component in surgical treatment planning because the patients’ perspective directly reflects the impact of surgical interventions on their everyday lives. Use of PROMs can facilitate the identification of potential underrated complications and enhance patient-centered care.

Despite the growing recognition of PROMs in surgery, a notable lack of validated, perineal-specific PROM instruments remains, as well as a scarcity of comparative outcome studies^24,25^ although these patients have a significant burden of restrictions after surgery in a deeply personal and intimate area of the body.^26^ This underscores the importance of reconstructive interventions, which aim not only to optimize surgical success but also to improve patients’ well-being and daily functioning. Consequently, this study addressed this gap by providing a systematic assessment of both surgical outcomes and patient-reported QoL after perineal reconstructive surgery using perforator versus non-perforator flaps.

## Material and Methods

### Study Design and Recruitment

In this single-center cohort study, a retrospective chart review was performed in conjunction with evaluation of postoperative patient-reported outcome measures. This study complied with the Declaration of Helsinki^27^ and was performed in accordance with the local ethics committee (“Ethikkommission Nordwest und Zentralschweiz (EKNZ)”: BASEC-ID: 2023-02296).

**Fig. 1:**
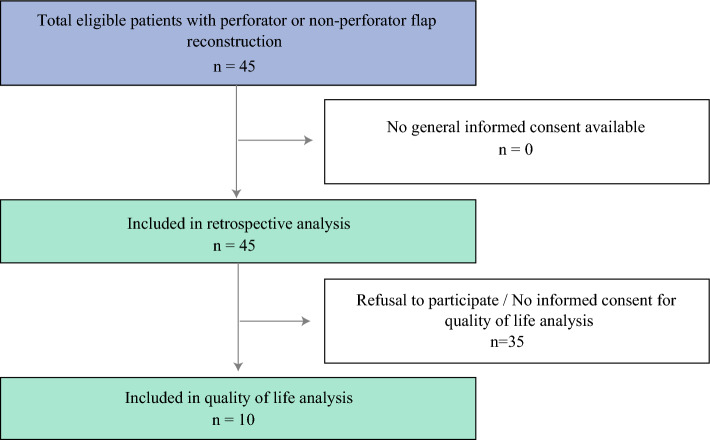
Flow chart of patient selection and study inclusion

The study included all medical records of patients who had undergone a perineal or anal reconstruction using either a perforator or a non-perforator flap between 2013 and 2023 at our tertiary care center. No formal sample size calculation was performed; the study size was determined by the number of available cases during this period. The inclusion criteria specified patients of any sex who were 18 years old or older, provided written informed and general consent, and had undergone perineal or anal reconstruction with either musculocutaneous, fasciocutaneous flaps or perforator flaps after surgical resection of oncologic, infectious, or inflammatory disease. The study ruled out all patients with perineal reconstruction secondary to congenital disorders or other reasons for genitourinary surgery, with primary closure of the defect, and/or without written informed or general consent. A flow chart is presented in Fig. 1.

Patients’ demographics and comorbidities were classified according to the American Society of Anesthesiologists (ASA) from ASA I (healthy) to ASA V (moribund).^28^ Details about the reconstructive procedure and postoperative complication were extracted from existing medical records and transferred into our electronic Case Report Form (eCRF) using the research electronic data capture (REDCap, Vanderbilt University Medical Center, Nashville, Tennessee, USA) software.^29,30^ For the postoperative evaluation of PROMs, all included patients were contacted by phone and informed about the study details. After signing the written informed consent, they completed the validated questionnaires.

An overview of the study’s key elements can be found in Table 1. To minimize potential sources of bias, this study relied on standardized clinical documentation. Consistent application of predefined inclusion and exclusion criteria helped reduce the risk of selection bias. The study considered PROMs only if completed in accordance with established guideline recommendations.^31–33^ Because all data were recorded before the analysis, the risk of observer bias was inherently limited.

**Table 1:** Overview of study characteristics

Patients	All adult patients with a flap-based reconstructive surgery in the pelvic/perineal region between 2013 and 2023
Intervention	Perforator flap-based perineal reconstruction
Comparison	Non-perforator flap-based perineal reconstruction
Outcome	Primary outcome: surgical complication rate Secondary outcome: patients’ satisfaction and PROMs
Study	Retrospective chart review with evaluation of postoperative PROMs

PROMs, patient-reported outcome measure

### The Concept of Non-Perforator Flaps

Non-perforator flaps have their blood supply from direct cutaneous vessels, eliminating the need for perforator vessel dissection. Usually there is a direct-axial-vessel or a random-pattern blood supply.^34^ These flaps consist of skin and subcutaneous fat tissue and may also include the underlying muscle. Usually when involving muscle, they become bulkier. Depending on the composition of the flap, it is classified either as a musculocutaneous or as a fasciocutaneous flap. Typically, non-perforator flaps are technically less demanding than perforator flaps, requiring fewer microsurgical skills.

Most defects in the perineal region can be managed with local and regional flaps.^35^ However, as defect size increases and large dead space volumes are present, a bulkier flap is required. In such situations, musculocutaneous flaps (e.g., VRAM), muscle flaps (e.g., gracilis), or fasciocutaneous flaps (e.g., gluteal-fold flap) are often required.^36–39^ Whereas muscle-based flaps such as the gracilis^40^ or vastus lateralis^41^ flaps often result in only minimal functional impairment, reconstructions involving larger muscles such as the VRAM are more likely to cause significant donor-site morbidity.^14,15,42,43^

### The Concept of Perforator Flaps

In the 1990s, Koshima et al.^21,44^ introduced the first perforator flap, the anteromedial thigh (ALT) flap, followed by the deep inferior epigastric (DIEP) flap, marking the beginning of a completely new flap type for reconstructive surgery. Perforator flaps involve the transfer of predominantly skin and subcutaneous tissue, supplied by isolated perforator vessels, usually without including full muscle components.^45^ These small perforator vessels originate from deeper arteries, transverse muscles, and fascia, ultimately perfusing a pre-defined area of the skin and subcutaneous fatty tissue. The dissection involves isolating these small vessels, going through muscle or fascia to supply the skin, allowing sparing of the muscle tissue.^21^ By using the perforator flap concept, donor-site morbidity can be greatly reduced while donor-site muscle function is preserved.^45^ Commonly used perforator flaps for perineal reconstruction are the profunda artery perforator flap (PAP) and the DIEP, ALT, and inferior and superior gluteal artery perforator (IGAP and SGAP) flaps.^46–52^

### Outcome Measures

The predefined primary endpoint was the rate of aggregate surgical complication occurring within the specified observational period. All complications were graded using the Clavien-Dindo classification, ranging from grade I to grade V.^53^

The secondary outcomes were patient-reported satisfaction with the postoperative result and QoL measured by various PROMs including the European Organization for Research and Treatment of Cancer (EORTC-C30) questionnaire, the female sexual function index (FSFI), the EuroQoL-5D-5L (EQ-5D-5L) instrument, and the EuroQoL-visual analog scale (EQ-VAS). The EORTC-C30 comprises the global health status, a functional scale, and a symptom scale. High scores in the global health status and functional scale represent a high level of functioning and a high QoL, but it represents a high level of symptomatology in the symptom scale.^33^ The FSFI captures different aspects of the sexual function in women including sexual desire, sexual activity, intercourse, and stimulation during the last 4 weeks ranging from “no activity at all” to “always.”^31^ The EQ-5D-5L measures health-related QoL ranging from 0 (worst) to 1 (best) and includes the EQ-VAS from 0 (worst imaginable health) to 100 (best imaginable health).^32^ Overall patients’ satisfaction was assessed on a 5-point Likert scale and dichotomized into “satisfied” (very satisfied, satisfied) and “unsatisfied” (neutral, unsatisfied, or very unsatisfied).

### Statistical Analysis

Descriptive statistics were used to summarize patient demographics, clinical characteristics, and outcome measures for both the perforator and non-perforator flap groups. Continuous variables were analyzed as means with standard deviations (SDs) or medians with interquartile ranges (IQRs), depending on data distribution. Categorical variables were reported as absolute count and percentages. To compare outcomes between the two groups (perforator vs non-perforator flaps), Fisher’s exact test was applied for categorical variables. For continuous variables, the Wilcoxon rank-sum test was used to compare both groups. A two-sided *p* value lower than 0.05 was considered statistically significant. Univariable linear regressions were performed separately for each group to explore potential associations. For most variables, missing data were minimal and handled with complete-case analysis. All statistical analyses were performed using the R statistical software (version 4.3.2; The R Foundation for Statistical Computing, Vienna, Austria).^54^

## Results

The study included 45 patients. Of these 45 patients, 18 (40 %) underwent non-perforator flap reconstruction, 26 (57.8 %) had perforator flap reconstruction, and 1 (2.2 %) received a combination of both. The mean follow-up period was 4.4 ± 3.5 years in the perforator flap group and 5.9 ± 3.5 years in the non-perforator flap group. No significant differences in age, body mass index (BMI), sex, or ASA classification were observed between the groups, as shown in Table 2. The leading indication for reconstructive surgery was malignancy, accounting for 31 cases (68.9 %). Additional underlying conditions comprised hidradenitis suppurativa (*n* = 5, 11.1 %) and infectious conditions (*n* = 8, 17.8 %). Nine patients (34.6 %) in the perforator flap group and seven patients (38.9 %) in the non-perforator flap group underwent adjuvant treatment including chemo-, radio-, or radiochemotherapy. The predominantly used flaps were the Singapore (28.6 %), PAP (20 %), and ALT (14.3 %) in the perforator flap group. In the non-perforator flap group, the most commonly used flap was the myofascial gracilis flap (38.1 %), followed by the fasciocutaneous gluteal rotational flap (28.6 %), as shown in Table 3.

**Table 2:** Patients’ characteristics between perforator and non-perforator flap-based reconstructions

	Perforator flap (*n* = 26) *n* (%)	Non-perforator flap (*n* = 18) *n* (%)		*p* value
Median age: years (IQR)	59.3 (56.7–69.0)	58.9 (46.0–64.5)	0.27	
Median BMI: kg/m^2^ (IQR)	24.9 (20.7–30.2)	25 (22.9–30.7)	0.558	
Sex			0.76	
Male	15 (57.7)	9 (50)		
Female	11 (42.3)	9 (50)		
ASA			0.504	
I	0 (0)	1 (5.6)		
II	5 (19.2)	4 (22.3)		
III	20 (76.9)	11 (61.1)		
IV	1 (3.9)	2 (11.1)		
Pre-existing noxae			1	
Nicotine abuse	13 (50)	9 (50)		
Surgical indication			0.035	
Hidradenitis suppurativa	1 (3.9)	4 (22.2)		
Anal cancer	3 (11.5)	3 (16.7)		
Extramammary Paget’s disease	1 (3.9)	2 (11.1)		
Fournier gangrene	5 (19.2)	3 (16.7)		
Rectal cancer	3 (11.5)	5 (27.8)		
Vulvar cancer	9 (34.6)	1 (5.5)		
Other^a^	4 (15.4)			
Adjuvant treatment				
Chemotherapy	0 (0)	1 (5.6)	0.409	
Radiotherapy	4 (15.4)	1 (5.6)	0.634	
Radiochemotherapy	5 (19.2)	5 (27.8)	0.716	

IQR, interquartile range; BMI, body mass index; ASA, American Society of Anesthesiologists classification

^a^Other includes vaginal cancer (*n* = 1), melanoma (*n* = 2), advanced seminoma (*n* = 1)

**Table 3:** Surgical characteristics between perforator and non-perforator flap-based reconstructions

	Perforator flap (*n* = 26) *n* (%)		Non-perforator flap (*n* = 18) *n* (%)		*p* value
Defect					0.303
Unilateral	17 (65.4)		15 (83.3)		
Bilateral	9 (34.6)		3 (16.7)		
Median defect size: cm^2^ (IQR)	123.2 (49–204)	97.5 (51–147)			0.450
Reconstructive methods	35 (100)			21 (100)	<0.001
Singapore	10 (28.6)		Gracilis	8 (38.1)	
PAP	7 (20)		Gluteal rotational flap	6 (28.6)	
ALT	5 (14.3)		VRAM	3 (14.3)	
Local rotational flap	4 (11.4)		V-Y	2 (9.5)	
DIEP	3 (8.6)		Posterior thigh flap	2 (9.5)	
IGAP	2 (5.7)		–	–	
SGAP	2 (5.7)		–	–	
Inguinal perforator flap	2 (5.7)		–	–	
Median surgical duration: min (IQR)	249 (165–375)		239.5 (134–384)		0.933
Median blood loss: ml (IQR)	105 (102.5–1500)		100 (100–800)		0.871
Donor-site closure					0.608
Primary closure	21 (80.8)		17 (94.4)		
Closure with mesh	1 (3.8)		1 (5.6)		
Split-thickness skin graft	2 (7.7)		0 (0)		
Local flap	2 (7.7)		0 (0)		

cm, centimeter; IQR, interquartile range; PAP, profunda artery perforator flap; ALT, anterolateral thigh flap; DIEP, deep inferior epigastric perforator flap; VRAM, vertical rectus abdominis musculocutaneous flap; ; IGAP, inferior gluteal artery perforator flap; SGAP, superior gluteal artery perforator flap; min, minutes; ml, milliliter

### Complications

In this study, 23 complications (51.1 %) were observed. Most (82.6 %) of the complications occurred at the recipient site, as shown in Table 4. The most frequent recipient-site complications were wound dehiscence (*n* = 7), partial flap necrosis (*n* = 5), and local infections (*n* = 2). Notably, no cases of total flap loss were observed. The overall complication rates were comparable between the two groups. Donor-site complications were more prevalent in the non-perforator flap group, exclusively observed in muscle-based reconstructions (VRAM, *n* = 2; gracilis, *n* = 1). Wound dehiscence (*n* = 2) and hematoma (*n* = 1) were predominant at the donor site. One postoperative hernia formation occurred in a patient with VRAM reconstruction, which required surgical repair. Most complications were classified as Clavien-Dindo grade IIIb, therefore necessitating surgical revision.

**Table 4:** Overall, recipient, and donor-site complications for perforator versus non-perforator groups

	Perforator flap (*n* = 26) *n* (%)	Non-perforator flap (*n* = 18) *n* (%)	*p* value
Overall complications	13 (50)	9 (50)	1
Recipient site			0.632
Clavien-Dindo grade I	3 (23.0)	2 (22.2)	
Clavien-Dindo grade II	2 (15.4)	0.00	
Clavien-Dindo grade IIIa	2 (15.4)	0.00	
Clavien-Dindo grade IIIb	5 (38.5)	4 (44.4)	
Donor site			*-*
Clavien-Dindo grade I	0.00	0.00	
Clavien-Dindo grade II	0.00	0.00	
Clavien-Dindo grade IIIa	0.00	0.00	
Clavien-Dindo grade IIIb	1 (7.7)	3 (33.3)	

Although the results were not statistically significant, this analysis identified numeric differences in complication risk across certain patient groups. Specifically, the patients undergoing bilateral reconstruction showed a numerically higher odds ratio (OR) of complications (OR, 1.56; 95 % confidence interval [CI], 0.42–6.39; *p* = 0.5). Similarly, the patients with a history of nicotine use demonstrated a threefold greater risk for the development of postoperative complications than non-smokers (OR, 3.06; 95 % CI, 0.92–10.94; *p* = 0.0742). Nicotine use was associated with a numerically higher odds ratio for complications in the non-perforator flap group (OR, 4.0; 95 % CI, 0.60–33.0; *p* = 0.166) than in the perforator flap group (OR, 2.56; 95 % CI, 0.54–13.3; *p* = 0.244). Additionally, an increased risk of complications was observed in patients with a BMI between 25 and 30 kg/m^2^ (OR, 1.94; 95 % CI, 0.42–10.91; *p* = 0.413). In contrast, no increased risk was observed for patients with a BMI greater than 30 kg/m^2^ or for those undergoing adjuvant cancer treatments (*p* > 0.165).

### Patient-Reported Outcome Measures and QoL

Based on responses from only 10 patients (22.2 %) who participated in the postoperative evaluation of PROMs, these findings are exploratory in nature. Of these patients, six (60 %) were from the perforator group and four (40 %) were from non-perforator flap group. The mean time between surgery and questionnaire was 1.7 ± 0.9 years in the perforator group and 4.1 ± 2.3 years non-perforator flap group. The majority of the patients (80 %) were satisfied with the postoperative result, with higher satisfaction reported by the non-perforator flap group. Nevertheless, overall, the collected PROMs were answered more favorably by the patients who underwent perforator flap-based reconstruction, as shown in Table 5. However, these differences did not reach statistical significance.

**Table 5:** Comparison of patient-reported outcomes after perforator versus non-perforator flap reconstruction

	Perforator flap (*n* = 6) *n* (%)	Non-perforator flap (*n* = 4) *n* (%)	*p* value
Satisfaction	6 (33.3)	4 (15.4)	0.467
Satisfied	4 (66.7)	4 (100)	
Unsatisfied	2 (33.3)	0 (0)	
EORTC-C30	6 (23.1)	4 (22.2)	
Median global health status/QoL (IQR)	58.3 (37.5–91.7)	41.7 (33.3–58.3)	0.524
Median functional scale (IQR)			
Physical functioning	70 (60–95)	53.3 (38.3–66.7)	0.314
Role functioning	66.7 (41.7–91.7)	41.7 (33.3–62.5)	0.729
Emotional functioning	58.3 (43.8–91.7)	20.8 (12.5–43.8)	0.205
Cognitive functioning	75 (54.2–95.8)	75 (58.3–83.3)	0.624
Social functioning	75 (54.2–95.8)	25 (16.7–41.7)	0.186
Median symptom scale (IQR)			
Fatigue	61.1 (13.9–66.7)	61.1 (27.78–91.7)	0.5
Nausea and vomiting	8.3 (0–29.2)	41.7 (25–54.2)	0.457
Pain	33.3 (8.3–70.8)	66.7 (50–70.8)	0.781
Dyspnea	0 (0–25)	16.7 (0–41.7)	0.69
Insomnia	16.7 (0–58.3)	83.3 (50–100)	0.381
Appetite loss	16.7 (0–33.3)	16.7 (0–50)	1
Constipation	0 (0–0)	16.7 (0–41.7)	0.133
Diarrhea	16.7 (0–58.3)	16.7 (0–41.7)	0.905
Financial difficulties	50 (0–100)	16.7 (0–50)	0.714
FSFI	3 (10.7)	2 (11.1)	
Median total score (IQR)	22.6 (15.3–25.5)	5.3 (4.8–5.7)	0.2
EQ-5D-5L	3 (10.7)	4 (22.2)	
Median EQindex score (IQR)	1 (0.78–1)	0.71 (0.5–0.9)	0.571
EQ-VAS	6 (23.1)	4 (22.2)	
Median total score (IQR)	67.5 (55.3–77.5)	61 (51.3–75)	1

EORTC-C30, European Organisation for Research and Treatment of Cancer; QoL, quality of life; IQR, interquartile range; FSFI, female sexual function index; EQ-5D-5L, EuroQoL-5D-5L; EQ-VAS, EuroQoL-visual analog scale

## Discussion

To the best of our knowledge, this is the first study to evaluate both complication rates and postoperative QoL in patients undergoing perineal reconstruction using either perforator or non-perforator flaps. Given the scarcity of data addressing the comparative surgical results of perforator and non-perforator flap perineal reconstruction and QoL outcomes in this context, this study addresses a significant gap in the current literature.

Perforator flaps are a valuable option in perineal reconstruction, offering reliable perfusion and reduced donor-site morbidity.^45^ Unlike musculocutaneous flaps, they consist primarily of subcutaneous fat, which is less prone to long-term atrophy than muscle tissue.^55–58^ Moreover, the possibility of flap sensibilization makes them particularly suitable for reconstruction in the perineal region,^59^ where sensory function plays an important role in patient QoL.

### Complication Rates after Perforator versus Non-Perforator Flap Reconstruction

An overall complication rate of 51.1 % was observed in this study, with the majority (82.6 %) of complications occurring at the recipient site. Previous studies have shown a comparable range of complications in patients undergoing flap reconstruction in the perineal region.^48,60–63^ Hellinga et al.^62^ performed perineal reconstruction using the lotus petal flap in 99 patients and reported an overall complication rate of 69.9 %, with 14 % occurring at the donor site. These findings are in line with the current study, which also demonstrated a high overall complication rate, predominantly at the recipient site.

Contributing factors to the high complication rate in this study included the high prevalence of patients with severe comorbidities (ASA III and higher, 75.5 %), a significant proportion of active smokers (48.9 %), and the inherent anatomic and microbiologic challenges of the perineal region, particularly its proximity to the anus, urethra, and genitals, which contribute to increased bacterial colonization and therefore a higher risk of postoperative complications. Furthermore, numeric differences were observed in the odds ratios for complications associated with nicotine use, with higher estimates for patients undergoing non-perforator flap reconstruction (OR, 4.0) than for those receiving perforator flaps (OR, 2.56), although this difference was not statistically significant.

Previous studies have demonstrated that smoking is associated with significantly higher complication rates in both types of flap reconstruction.^64–66^ Microvascular vasoconstriction in smokers may impair tissue perfusion, particularly in cutaneous vessels and in tissues located at a greater distance from the main vascular supply.^67^ The reliable and predictable perfusion provided by the defined angiosome-based vascular territory of perforator flaps^20^ may mitigate the effects of nicotine-induced vasoconstriction, potentially explaining the lower odds ratios for complications observed in these flaps.^68^

Bilateral reconstruction, performed for 26.7 % of the patients in this cohort, was associated with an increased risk of postoperative complications compared with unilateral procedures (OR, 1.56). This may reflect the combination of existing risk factors and the compound burden of multiple surgical sites with a consequently larger defect area and exposed anatomic structures.

Complications classified as Clavien–Dindo grade III, indicating requirement of surgical revision, accounted for 61.1 % at the recipient site and 100 % at the donor site. A greater number of conservatively manageable complications are observed at the recipient site, primarily due to superficial wound-healing disorders in the contaminated perineal region. In contrast, complications at the donor site often affect deeper tissue layers or lead to functional impairment, thereby making surgical intervention more likely.

Additionally, anatomic donor sites (abdomen, thigh, gluteal) are associated with distinct, site-specific morbidity profiles. In abdominal-based flaps, morbidity is primarily related to impairment of the abdominal wall, including weakness, with potential hernia or bulge formation, residual strength deficits, and local wound complications.^69^ In contrast, thigh-based flaps tend to shift the complication spectrum toward local wound-healing problems and sensory disturbances related to peripheral nerve irritation or injury.^70,71^ For gluteal-based flaps, contour changes, sitting-related discomfort, local wound complications, seroma formation, and delayed sensory recovery represent key donor-site considerations.^72–74^

In light of the heterogeneity of anatomic donor sites used, each with distinct morbidity profiles, it is possible that these anatomic differences influenced the overserved complication rates independently of perforator versus non-perforator classification. Importantly, given the inherently high overall complication burden of perineal reconstruction, strategies to minimize additional donor-site morbidity remain a critical factor in flap selection and preoperative patient counselling.

### Difference in PROMs Between Perforator and Non-Perforator Flaps

The standardized assessment of patients’ QoL is of utmost importance to ensure continuous improvement of surgical techniques and to achieve the best possible outcomes for individual patients. The aim of this retrospective cohort study was to compare the patient-reported QoL after perforator and non-perforator flap perineal reconstruction. Although the results were not statistically significant, were exploratory in nature, and were based on questionnaire responses from only 10 patients, descriptive PROM data showed numerically higher EORTC-C30, FSFI, and EQ-5D-5L scores for patients undergoing perforator flap reconstruction (Table 5). These findings are descriptive and do not support inferential conclusions regarding differences in QoL. Although patient-reported satisfaction was greater after non-perforator flap reconstruction (100 % vs 66 %), this finding should be interpreted with caution because postoperative QoL and satisfaction in the perforator flap group were measured considerably earlier (1.74 vs 4.14 years after surgery). Previous research has demonstrated that both QoL and postoperative satisfaction tend to improve over time after reconstructive surgery.^75,76^ Therefore, the temporal difference in follow-up findings may have a positive impact on the perceived outcomes of perforator flap reconstruction if assessed during a longer postoperative period.

To the best of our knowledge, no previous study has evaluated PROMs comparing perforator and non-perforator flap reconstruction in the context of perineal reconstruction, making it difficult to compare our findings directly with those of others. In addition, the field lacks dedicated PROMs specifically addressing the perineal region after reconstructive procedures,^25^ in contrast to breast surgery, in which validated instruments such as the Breast-Q have become an integral part of outcome evaluation.^77^ Implementing a standardized questionnaire alongside systematic collection of PROMs may optimize flap-based perineal reconstruction and contribute to improved patient QoL. In the sensitive and intimate anatomic region of the body, PROMs should serve as a central factor in advocating for specific surgical approaches, particularly when favorable postoperative QoL is reported.

### Strength and Limitations

This study provided the comparison of surgical outcomes with a mean follow-up duration of 4.40 years in the perforator flap group and 5.94 years in the non-perforator group. Postoperative satisfaction and PROMs were likewise obtained after substantial postoperative periods of 1.74 and 4.14 years, respectively, underscoring the strength of the long-term perspective in evaluating patient-centered outcomes.

Despite these strengths, several limitations must be acknowledged when the results of this study are interpreted. One key limitation of this study was the small sample size, which restricted the ability to achieve statistical significance. This limitation may be explained by the fact that the underlying conditions requiring reconstruction are primarily managed within visceral surgery and gynecology. Reconstructive plastic surgeons are typically involved only in selected, particularly complex cases for which their specialized expertise is especially valuable. Consequently, the number of cases in this specific reconstructive context remains limited despite the high relevance and potential benefit of plastic surgical involvement. In a large cohort, it would ideally have been possible to perform a subgroup analysis comparing abdominal-, thigh-, and gluteal-based donor sites. Moreover, defect etiology and anatomic donor site-specific characteristics may independently influence donor-site morbidity and complication profiles beyond the perforator versus non-perforator classification. It is possible that one donor site offers clearly superior outcomes compared with others, which could be important in the counseling of patients for whom several anatomic options are available and feasible.

Another limitation of this study was the small number of patients included in the evaluation of PROMs, which may be attributable to its retrospective design and long follow-up period. Many patients could not be contacted due to the unavailability of current contact information. The unwillingness to participate in the postoperative collection of PROMs may be attributable to a desire among some patients to mentally dissociate from the burden of their illness, viewing further involvement as a reminder of a distressing period.

In addition, the lack of baseline PROM data further complicates the interpretation of how reconstruction affected QoL. Moreover, the significant difference in follow-up time between the two groups (1.7 vs 4.1 years) introduced a potentially relevant confounding factor because satisfaction and QoL scores are known to improve over time.^75,76^ These limitations highlight the importance of implementing standardized PROM collection from the beginning of treatment in future studies.

## Conclusion

The perineal region represents a highly complex anatomic and functional area in which reconstructive procedures are inherently associated with considerable complication rates irrespective of whether perforator or non-perforator flaps are used or not. However, perforator flap techniques offer the advantage of reducing donor-site morbidity, thereby minimizing additional patient burden. At the same time, given the anatomic heterogeneity of donor sites, observed donor-site morbidity may partly reflect region-specific anatomic characteristics beyond perforator versus non-perforator classification. Although long-term outcomes remain part of further investigations, preliminary PROMs suggest a potential signal toward improved QoL for patients undergoing perforator flap reconstruction, acknowledging the exploratory nature of these findings.

These findings underscore the need for standardized, region-specific PROMs and long-term follow-up studies to better evaluate patients’ QoL and guide future reconstructive strategies in the perineal region. We believe that the assessment of PROMs should be part of the standardized follow-up protocols in all institutions. Furthermore, closer interdisciplinary collaboration between reconstructive surgery, visceral surgery, and gynecology may lead to improved long-term outcomes focused on patient well-being.

## Data Availability

The data that support the findings of this study are not publicly available pursuant to the ethics committee approval by the “Ethikkommission Nordwest und Zentralschweiz EKNZ” in Switzerland. The data can be obtained from the corresponding author upon reasonable request.
